# Immunogenicity of HIV-1 Vaccines Expressing Chimeric Envelope Glycoproteins on the Surface of Pr55 Gag Virus-Like Particles

**DOI:** 10.3390/vaccines8010054

**Published:** 2020-01-29

**Authors:** Rosamund Chapman, Michiel van Diepen, Shireen Galant, Elizabeth Kruse, Emmanuel Margolin, Phindile Ximba, Tandile Hermanus, Penny Moore, Nicola Douglass, Anna-Lise Williamson, Edward Rybicki

**Affiliations:** 1 Institute of Infectious Disease and Molecular Medicine, Faculty of Health Science, University of Cape Town, Cape Town 7925, South Africa; 2 Division of Medical Virology, Department of Pathology, University of Cape Town, Cape Town 7925, South Africa; 3 Division of Biomedical Engineering, Department of Human Biology, University of Cape Town, Cape Town 7925, South Africa; 4 Biopharming Research Unit, Department of Molecular and Cell Biology, University of Cape Town, Cape Town 7701, South Africa; 5 Centre for HIV and STIs, National Institute for Communicable Diseases of the National Health Laboratory Service, Johannesburg 2131, South Africa; 6 Antibody Immunity Research Unit, Faculty of Health Sciences, University of the Witwatersrand, Johannesburg 2050, South Africa; 7 Centre for the AIDS Programme of Research in South Africa (CAPRISA), University of KwaZulu-Natal, Congella, Durban 4013, South Africa

**Keywords:** HIV-1, vaccine, chimeric, VLP, spike density, envelope

## Abstract

The HIV-1 envelope glycoprotein (Env) is present on the surface of the virion at a very low density compared to most other enveloped viruses. Substitution of various parts of the stalk domain of Env (gp41) with the corresponding elements from other viral glycoproteins has been shown to increase Env spike density on the cell membrane and surface of virus-like particles (VLPs). In this study, chimeric Env antigens were generated by replacing the transmembrane and cytoplasmic domains of HIV-1 Env with the corresponding regions from the influenza H5 hemagglutinin (HA) (gp140HA_2_tr) and by replacing the entire gp41 region of Env with the HA_2_ subunit of HA (gp120HA_2_). Recombinant DNA and modified vaccinia Ankara (MVA) vaccines expressing HIV-1 subtype C mosaic Gag and gp150 Env or either of the chimeras were generated. Surprisingly, no significant differences were found in the levels of expression of gp150 Env or either of the chimeras on the surface of cells or on Gag VLPs. Differences were, however, observed in the binding of different monoclonal antibodies to the HIV-1 Env. Monoclonal antibodies, which recognized a V1 / V2 quaternary epitope at the tip of the native Env trimer, bound gp150 and gp140HA_2_tr chimera but failed to bind to the gp120HA_2_ chimera. Autologous Tier 2 neutralizing antibodies (NAbs) were produced by rabbits inoculated with DNA and MVA vaccines expressing the gp140HA_2_tr chimera or gp150 Env, but not those immunized with the gp120HA_2_ Env. These results showed that the addition of an HA_2_ stalk to HIV-1 gp120 did not improve immunogenicity, but rather that the full-length gp150 was required for optimal presentation of epitopes for the elicitation of a neutralizing antibody response to HIV-1.

## 1. Introduction

Although there has been a reduction in deaths related to HIV infection in recent years due to the implementation of antiretroviral therapy as well as other measures, the AIDS pandemic continues to grow. Approximately 37 million people are living with HIV today, and about 1.8 million became newly infected in 2019 [1]. In 2017, sub-Saharan Africa accounted for nearly 65% of new infections globally. The most effective way to control this pandemic is the development of a prophylactic HIV-1 vaccine. It is thought that an HIV envelope (Env) immunogen that can either elicit broadly neutralizing antibodies (bNAbs) that prevent HIV infection or one that elicits polyfunctional, non-neutralizing antibodies that drive the clearance of the virus are possible approaches to generating a prophylactic HIV vaccine [2].

The envelope glycoproteins of most viruses are present in dense (50–100Å), repetitive arrays on the surface of the virion [3]. This highly ordered, dense spacing of epitopes is not often found on the surface of mammalian cells and is thus thought to be a key determinant of recognition by the humoral immune system of viruses as being foreign [4]. The binding and subsequent cross-linking of the B cell receptors on the surface of naïve B cells by these highly ordered repetitive antigens strongly activates B cells, promoting high-titer, durable antibody responses [4,5,6]. HIV-1, however, has an unusually low density of envelope (Env) spikes (7–14 spikes / virion) on its surface [3,7]. In comparison, influenza virions have 400 to 500 spikes per similar-sized virion.

Several groups have made modifications to the HIV-1 Env protein to increase the density on the surface of virus-like particles (VLPs). The fusion of the HIV-1 gp120 protein to the Epstein–Barr virus gp220 / 350-derived transmembrane region (TM) resulted in 10 times higher incorporation into Gag VLPs than the wild type HIV-1 gp160 envelope protein [8]. Substitution of HIV Env signal sequence with that of the honeybee melittin protein (HMSS) and the TM and cytoplasmic tail (CT) with those of the mouse mammary tumor virus, influenza virus hemagglutinin, or baculovirus gp64 envelope glycoproteins increased incorporation of Env in Gag VLPs by up to 14 fold [9,10]. Similarly, a stable insect cell line constitutively expressing HIV Gag VLPs presenting a chimeric envelope protein containing the HMSS, HIV gp140, and baculovirus gp64 TM and CT with SOSIP stabilizing mutations resulted in 6–12 fold higher Env:Gag ratios than observed with native HIV-1 virions [11].

A non-toxic variant of vesicular stomatitis virus (VSV), known as VSV-GP, has been constructed in which the glycoprotein (G) from VSV has been replaced with the glycoprotein (GP) of the lymphocyte choriomeningitis virus. VSV-GP has been used as a vector to compare the full-length HIV Env and chimeras of HIV gp140 fused to the TM + CT region of the VSV glycoprotein G, with an intact or mutated furin cleavage site or a glycine linker sequence in place of the furin cleavage site (VSV-GP-gp140:G-linker) [12]. No difference was observed in HIV Env density on the surface of cells infected with the different VSV-GP vectors, but the HIV Env/VSV-G chimeras were incorporated into the VSV-GP virus particles better than full-length HIV Env.

Although HIV-1 chimeras have been shown to incorporate better into VLPs, it is not known whether these chimeras have the native-like trimeric conformation that is recognized by many bNAbs. In addition, most groups have not investigated the immunogenicity of these chimeras, specifically with regard to their ability to induce neutralizing antibodies. Bresk et al. 2019 [12] found that bNAb PG16, which binds to a native, trimeric epitope at the V2 apex of gp120, bound to the VSV-GP-gp140:G-linker chimera poorly, and rabbits vaccinated with the recombinant virus did not develop any Tier 2 NAbs. Similarly, the chimeric envelope protein, consisting of the HMSS, HIV-1 gp140, and TM and CT of baculovirus gp64, was shown to bind monoclonal antibodies (MAbs) 2G12, b12, 2F5, and 4E10 in a Western blot but elicited poor neutralizing antibody responses in mice [11].

In this study, the native HIV envelope signal sequence was replaced with the human tissue plasminogen activator (TPA) leader sequence, and two chimeras were made. In the first chimera, the HIV gp120 and gp41 ectodomain were retained, and the TM and CT were replaced with that of the influenza hemagglutinin protein HA_2_. In the second chimera, the entire HIV gp41 was replaced with the influenza hemagglutinin HA_2_, which is the analogous transmembrane subunit of the glycoprotein. DNA and modified vaccinia Ankara (MVA) vaccines expressing these chimeras were constructed, and the structure of the chimeric envelope proteins expressed on the surface of cells transfected/infected with these vaccines was assessed using different monoclonal antibodies. All the vaccines constructed were shown to express Gag VLPs containing Env or Env chimeras. The immunogenicity of the DNA and MVA vaccines expressing the two chimeras was compared to that of DNA and MVA vaccines expressing gp150 (also with a TPA leader sequence, as previously described in van Diepen et al. 2019 [13]).

## 2. Materials and Methods

### 2.1. Antibodies, Plasmids, Cell Lines, Media, and Reagents

Goat anti-HIV-1 gp160 (MRC ADP 72 408/5104), rabbit anti-HIV-1 p24 (Gag) (ARP 432), donkey anti-goat IgG Cy3 or FITC, and donkey anti-rabbit IgG Alexa 647 (Life Technologies, Oslo, Norway) were used for immunofluorescence assays. Goat anti-human IgG-Cy3 (Fc-specific) antibody (Sigma, Saint Louis, Missouri, USA) was used for live-cell staining and fluorescence-activated cell sorting (FACS) assay. Goat anti-HIV-1 gp120 (BioRad 5000-0557, Bio-Rad, Watford, UK), goat anti-HIV-1 p24 (Gag) (BioRad 4999–9007, Bio-Rad, Watford, UK), and mouse monoclonal anti-goat/sheep IgG–AP GT34 (Sigma, Saint Louis, Missouri, USA) were used for Western blotting. Anti-HIV-1 Env human monoclonal antibodies (MAbs) PG9, PG16, PGT128, PGT135, PGT145, CAP256-VRC26.08, VRC01, 10E8, 447-52D, and F105 were expressed in FreeStyle 293F cells (Life Technologies, Oslo, Norway) using the PEIMAX transfection reagent (Polysciences, Warrington, Pennsylvania, USA). Monoclonal antibodies were purified from cell-free supernatants after 6 days using Protein-A affinity chromatography [14]. CAP256 SU V1V2 scaffolded proteins were produced and purified by affinity chromatography using a Ni-NTA column, as described previously by Gorman et al. (2016) [15].

### 2.2. Design and Construction of DNA and MVA Vaccines Expressing Chimeric Env

**Figure 1:**
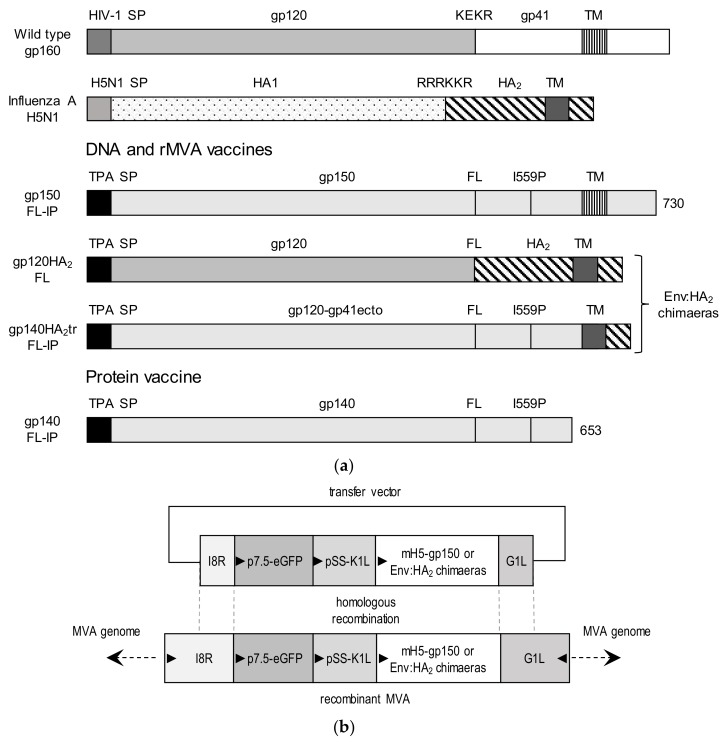
Design of DNA and modified vaccinia Ankara (MVA) vaccines expressing the chimeric envelope antigens. (**a**) Schematic representations of wildtype gp160, influenza hemagglutinin (HA), gp150-FL-IP, gp120HA_2_-FL, gp140HA_2_tr-FL-IP, and gp140-FL-IP. The gp160 sequence was truncated at amino acid residue 730 to generate gp150, the native signal peptide sequence (SP) was replaced with the human tissue plasminogen activator leader sequence (TPA), the furin cleavage motif, KEKR, was replaced with a flexible linker (FL), and an I559P (I-P) mutation was introduced. The soluble protein, Gp140-FL-IP, was truncated at amino acid 653. (**b**) Schematic representation of transfer vector for targeting Env-HA_2_ chimeras, expressed by the mH5 promoter, into the I8R-G1L locus of MVA-Gag^M^. Triangles indicate the direction of open reading frames.

The sequence of the CAP256 SU gp160 (clone CAP256.206sp.032.C9) has been previously described (GenBank: KF241776.1) [16]. The Env sequence was altered, as previously described, to improve expression and trimer formation. Briefly, the native leader sequence was replaced with the TPA leader peptide, the furin cleavage site was replaced with flexible linker (FL) consisting of 2 repeats of (GGGGS)_2_, and an isoleucine was mutated to proline (I559P) in the gp41 region, as described previously (van Diepen, Chapman et al. 2019). Two different chimeras were generated in which either the whole of the HIV-1 gp41 subunit was replaced with the corresponding influenza HA_2_ stalk (gp120HA_2_-FL), or the ectodomain of gp41 (including the I559P mutation) was retained and the transmembrane domain and cytoplasmic tail replaced with that of HA_2_ (gp140HA_2_tr-FL-IP) (see Figure 1a). The HA_2_ stalk sequence was derived from influenza A H5N1 strain (NC_007362.1). For all constructs, any potential poxvirus termination signals (TTTTTNT) were removed from the coding sequence, and a poxvirus termination signal was added directly after the stop codon of the envelope gene. The chimeric Env sequences were human codon-optimized and synthesized by GenScript (Hong-Kong). The mammalian expression plasmid pTHPcapR [17] was used to construct DNA vaccines pMExT CAP256 gp150-FL-IP, pMExT CAP256 gp120HA_2_-FL, and pMExT CAP256 gp140HA_2_tr-FL-IP. The DNA vaccine expressing the HIV-1 subtype C mosaic Gag, pTJDNA4, has been described previously [18]. All DNA vaccines were synthesized by Aldevron (Fargo, North Dakota, USA).

Transfer vectors were designed to insert the chimeric envelope genes, under the control of the VACV mH5 promoter, into the genome of MVA-Gag^M^ [19]. The coding sequences were targeted between the G1L and I8R transcriptionally convergent open reading frames (ORFs) (Figure 1b). The enhanced green fluorescent protein (eGFP) gene expressed from the vaccinia virus (VACV) p7.5 promoter and the K1L host range gene expressed by the pSS promoter were used as marker and selection genes, respectively. Recombinant MVAGC2 (CAP256 gp120HA_2_-FL) and MVAGC4 (CAP256 gp140HA_2_tr-FL-IP) were constructed, as described previously [13]. The MVA, described in van Diepen et al. 2019 [13], expressing CAP256 gp150–FL-IP and Gag^M^, was referred to as MVAGC5 in this publication.

Putatively trimeric soluble CAP256 SU gp140 envelope protein (gp140 FL-IP) was prepared and characterized, as previously described [13].

### 2.3. Confirmation of Gag and Chimeric Env Expression by DNA and MVA Vaccines

Transfections of HEK293T cells were performed in 4 Well Permanox^®^ Slides (Lab-Tek^®^, Brendale, Australia) for immunofluorescent staining, using 1µg of plasmid DNA (0.5µg Env or Env:HA_2_ chimeras + 0.5µg Gag^M^) and 3µL X-tremeGENE (Roche, Mannheim, Germany). HEK293T cells were infected at a multiplicity of infection (MOI) of 0.5 with MVA vaccines. Western blotting and immunofluorescent staining of cells were carried out, as described in van Diepen et al. 2019 [13].

### 2.4. Live Cell Staining with Human, Monoclonal Antibodies to HIV-1 Env

Immunostaining of HeLa cells transfected with plasmid DNA or infected with rMVA was conducted with human monoclonal antibodies (MAbs) to assess the structural integrity of the recombinant Env antigens, as described previously [13]. To quantify the binding of MAbs to gp150 and the chimeric envelope proteins on the plasma membrane, a FACS assay, described by Samal *et al*., 2019, was adapted [20]. In short, transfections of HEK293T were performed in T75 flasks using 30µg of plasmid DNA and 90µL polyethyleneimine-branched (PEI). Three days after transfection, confluent cell layers were dislodged using a cell scraper, washed 3 times (475g spin for 4 min at room temperature) with buffer 1 (DMEM + 5% FCS), and the cell pellets were reconstituted in buffer 1. Cells were transferred to a 96-well round bottom plate and incubated with MAbs (final concentration of 10µg/mL) for 1 h at room temperature. After three washes with buffer 1, cells were incubated with anti-Human IgG-Cy3 (Fc specific) antibody (1:500 in buffer 1) for 1 h at room temperature. Cells were then washed three times with buffer 2 (5% FCS in PBS) before fixing in buffer 2 + 0.5% formaldehyde. Cells were analyzed on a FACS Canto II Analyzer (BD Biosciences), measuring FSC (forward scatter), SSC (side scatter), and Cy3 intensity. A total of 100,000 cells were acquired per MAb with BD FACSFlow buffer as the sheath fluid. Data were analyzed using the FlowJo software by gating cells and avoiding debris and determining the mean fluorescent intensity (MFI) of Cy3. For each MAb, untransfected cells were used as a control to determine the Cy3 negative and positive cell populations after FSC / SSC correction. To compare the gp150 and chimeric envelope constructs, an Env score for each MAb was calculated (MFI × Cy3 positive cells)/(Cy3 positive + negative cells).

### 2.5. VLP Isolation and Characterization

Transfections of HEK293T cells were performed in T150 flasks using 60µg of plasmid DNA (30µg Env or EnvHA_2_ chimeras + 30µg Gag^M^), and 180µL PEI. HEK293T cells were infected at an MOI of 0.5 with MVA vaccines. VLP isolation to determine Env:Gag ratios by Western blotting was performed using two sequential OptiPrep (Sigma, Saint Louis, Missouri, USA) density gradient ultracentrifugation steps, as described previously [13].

In order to visualize VLPs on EM grids, HEK293T cells were transfected in T75 flasks using 30µg of plasmid DNA (15µg Env or EnvHA_2_ chimeras + 15µg Gag^M^) and 90µL PEI. After 72 h, VLPs were filtered through a 0.2µm filter and then pelleted through a 5 mL 12% OptiPrep (in TBS) cushion. The samples were centrifuged at 50,228g, 4°C, with no break for 1 h. Pellets containing VLPs were reconstituted in 100µL TBS + 20% glycerol. Freshly activated carbon grids were coated with VLP preparations, stained with uranyl acetate, and imaged on a FEI/Tecnai T20 transmission electron microscope (TEM).

### 2.6. Rabbit Immunizations

Female New Zealand White rabbits were housed in the animal facility of the Faculty of Health Sciences at the University of Cape Town (UCT). Groups of 4 to 5 rabbits were used. All the animal procedures were approved by the UCT Animal Research Ethics Committee (reference UCT AEC 014–030 and 015–051) and performed by trained animal technologists. DNA and MVA vaccines were administered intramuscularly into the hind leg at 100µg (100µL of each) and 10^8^pfu (500µL), respectively. Protein was given as an intramuscular injection of 40µg of CAP256 SU gp140 in 500µL 1:1 (v/v) AlhydroGel^®^ (InvivoGen, Toulouse, France).

### 2.7. Anti-CAP256 SU gp140 and Scaffolded-CAP256 SU V1V2-loop Enzyme-Linked Immunosorbent Assay (ELISA)

To assess Env binding antibody titers in rabbit sera, binding ELISAs were performed. Plates were coated overnight with 10 ng/well CAP256 SU gp140 protein [13]. Rabbit sera were used in the primary incubation in a serial dilution range starting at 1:10. PBST (PBS containing 0.1% Tween 20 (Sigma, Saint Louis, Missouri, USA) was used instead of PBS for all subsequent steps. The detection antibody was goat anti-rabbit IgG-HRP conjugate (1:10,000) (Roche, Basel). ELISAs for the whole time course and each group were performed at the same time on duplicate plates. Duplicate data points were averaged and fitted to a non-linear regression curve (log(agonist) vs. response -- variable slope) in GraphPad Prism 5.0. Antibody end-point titers (X) were calculated as follows: (Y = Bottom + (Top-Bottom)/(1 + 10^((LogEC50 − X) * HillSlope))), with the threshold (Y) set as: (week 0 Bottom + (2 * standard error of week 0 Bottom)) for each time point. Data were plotted as the mean +/- SEM for the whole group. Binding ELISAs to CAP256 SU V1V2 loop scaffolded protein [21] for week 0 and 2 weeks after the final MVA inoculation or protein boost were performed in a similar fashion, but plates were coated with 500ng / well of protein.

### 2.8. HIV Neutralization Assays

Neutralizing antibody titers were determined using the standardized TZM-bl pseudovirus neutralization assay, as described previously [22].

### 2.9. Statistical Analysis

All statistical analysis was performed using GraphPad Prism 5.0 (San Diego, California, USA). Both one-way and two-way ANOVA were performed with Bonferroni post hoc testing.

## 3. Results

### 3.1. Design of Chimeric Env Proteins

To determine the effect of exchanging portions of the HIV gp41 protein with the analogous regions from influenza HA_2_ on the incorporation of HIV Env into Gag VLPs, two chimeras were constructed (Figure 1a). In the first chimera, the HIV gp120 and gp41 ectodomain were retained, and the TM and CT were replaced with that of the influenza hemagglutinin protein HA_2_ (gp140HA_2_tr-FL-IP). In the second chimera, the entire HIV gp41 was replaced with the influenza hemagglutinin HA_2_ subunit (gp120HA_2_-FL). The chimeras were compared to HIV gp150-FL-IP. In all three antigens, the natural leader sequence was replaced with the TPA leader, and the furin cleavage site with a flexible linker (FL) [23]. An I559P mutation (IP) [24] was included in gp150-FL-IP and gp140HA_2_tr-FL-IP to improve the trimerization of gp41. DNA vaccines that expressed these antigens were constructed using the pTHPCapR plasmid, which contains a porcine circovirus enhancer element known to significantly increase antigen expression [17]. Recombinant MVA was constructed by inserting the chimeric envelope genes into MVA-Gag^M^ [19] between the G1L and I8R ORFs under the control of the mH5 promoter (Figure 1b).

### 3.2. Expression of Chimeric HIV Envelope Proteins

**Figure 2:**
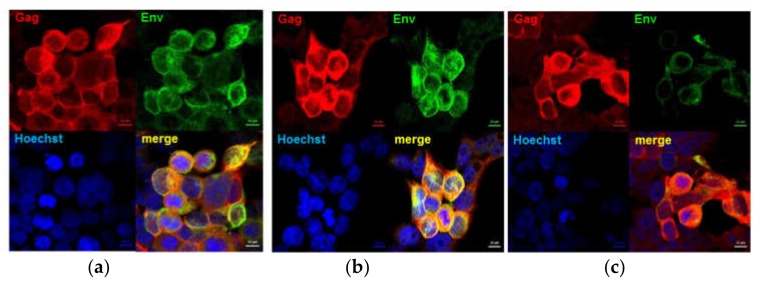
Confirmation of expression of chimeric envelope proteins and Gag. Confocal images of HEK293T cells infected with MVA show that both Env (green, Cy3) and Gag (red, Alexa Fluor 647) were expressed by all three vaccines. (**a**) MVAGC5 (Gag^M^ + gp150), (**b**) MVAGC2 (Gag^M^ + gp120HA_2_), and (**c**) MVAGC4 (Gag^M^ + gp140HA_2_tr).

Expression of Gag and each of the envelope proteins in cells co-transfected with the DNA vaccines or infected with the MVA vaccines was confirmed by Western blotting (results not shown) and confocal microscopy (Figure 2). Gag and Env were also detected in the cell media by Western blot analysis, confirming that the proteins were all secreted.

**Figure 3:**
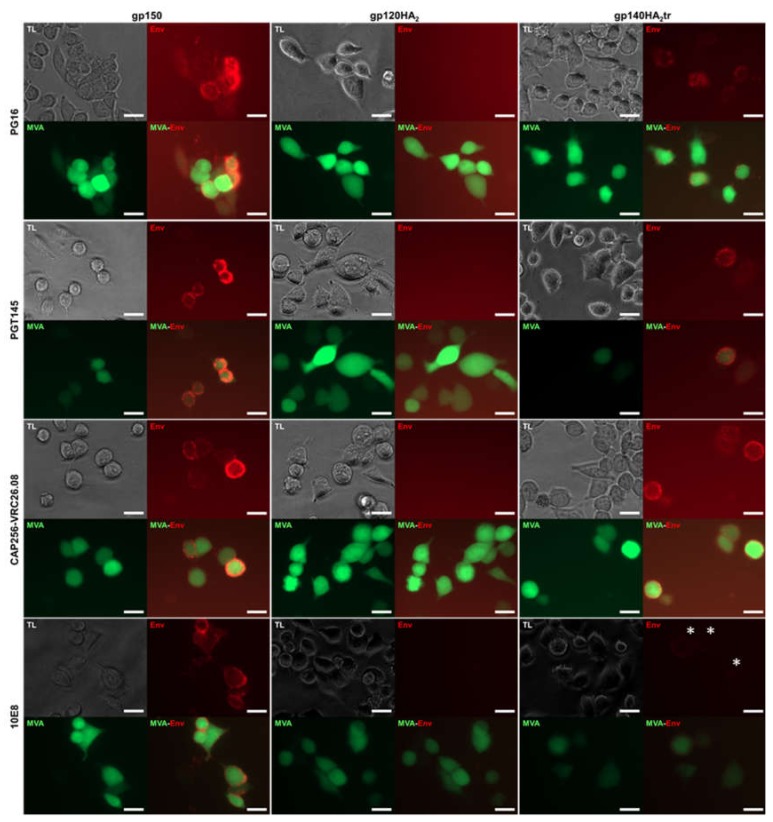
Characterization of the envelope chimeras expressed on the surface of the cell. HeLa cells infected with MVAGC5 (Gag^M^ + gp150), MVAGC2 (Gag^M^ + gp120HA_2_), and MVAGC4 (Gag^M^ + gp140HA_2_tr) were stained with monoclonal antibodies (MAbs) PG16, PGT145, CAP256-VRC26.08 (which all bind quaternary epitopes found on native, trimeric Env) and 10E8 (which binds to the membrane-proximal external region (MPER) of gp41). TL = transmitted light, phase contrast. MAbs were detected with anti-human IgG-Cy3 (red), and cells infected with MVA were visualized by their eGFP expression (green). Scale bars represent 20 µm. White stars indicate a weak Cy3 (red) signal for binding of MAb 10E8 to cells expressing gp140HA_2_tr.

**Figure 4:**
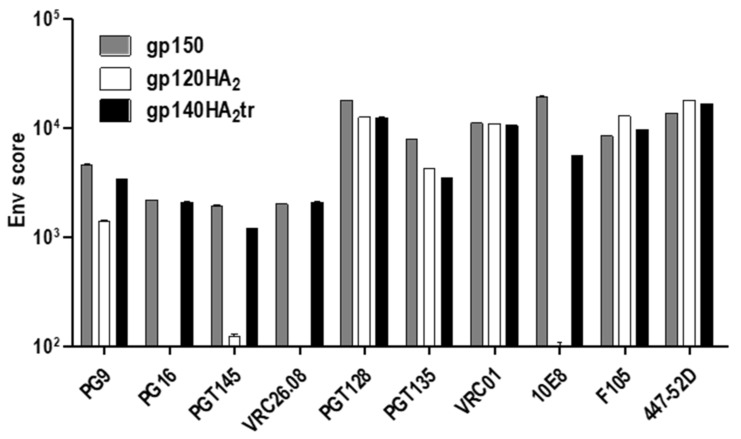
Differential binding of MAbs to gp150, gp120HA_2_, and gp140HA_2_tr. FACS-based cell surface staining of HEK293T cells, transfected with DNA vaccines expressing gp150, gp120HA_2_, and gp140HA_2_tr, with bNAbs and non-NAbs F105 and 447-52D. VRC26.08 = CAP256-VRC26.08.

The quaternary structures of gp150 Env and the chimeric Env proteins on the cell surface were assessed by immunostaining with human monoclonal binding antibodies (MAbs) to HIV-1 Env and fluorescent microscopy or FACS analysis (Figure 3; Figure 4). All three vaccine-expressed Envs bound neutralizing MAbs PGT128, PGT135 (V3 glycan), VRC01 (CD4 binding site), PG9 (V1 / V2), and non-neutralizing MAb F105 (CD4 binding site), which do not differentiate between native, trimeric, and non-native Env (Table 1). However, only gp150 and gp140HA_2_tr bound MAbs PG16, PGT145, and CAP256-VRC26.08, which bind to the quaternary V1/V2 epitope at the tip of the native Env trimer (Figure 3 and Table 1). Similarly, binding of MAb PG9 to gp120HA_2_ was slightly lower than to gp140HA_2_tr and gp150. PG9 does show some preference for native, trimeric Env but not as much as PG16 [25,26]. This indicates that the gp120HA_2_ chimera was not displayed in a native, trimeric structure on the cell surface. In addition, gp120HA_2_ showed higher levels of binding to MAb F105 than Gp150 and gp140HA_2_tr, indicating it was displayed on the surface of the cell in a more non-native-like conformation than the other two antigens (Figure 4). This epitope is sequestered in the interior of well-ordered Env trimers [27]. As expected, there was no binding of MAb 10E8 to the gp120HA_2_ chimera as this antibody binds to the membrane-proximal external region (MPER) of HIV Env, which has been replaced with the influenza HA_2_ region. All three Env antigens showed similar levels of binding to the V3-specific, non-neutralizing MAb, 447-52D. There was little difference in the binding of antibodies that recognize epitopes specific for native Env trimers between Gp150 and gp140HA_2_tr, but Gp150 showed slightly increased binding of PGT128, PGT135, and 10E8 (Figure 4). In addition, Gp150 showed decreased binding of F105 in comparison to both gp140HA_2_tr and gp120HA_2_.

### 3.3. Characterization of VLPs

**Figure 5:**
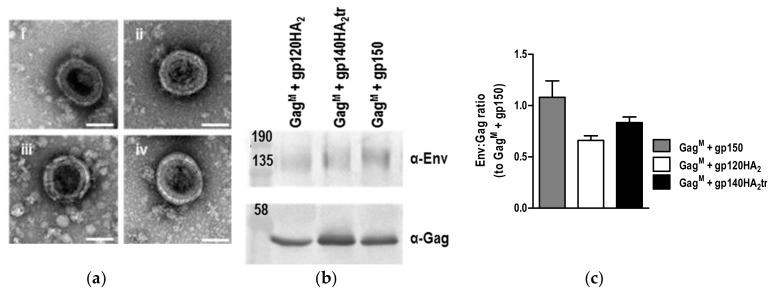
Analysis of virus-like particles (VLPs) formed by the DNA and MVA vaccines. (**a**) Negative staining of VLPs formed by HEK293T cells transfected with DNA vaccines expressing (**i**) Gag^M^ + gp150, (**ii**) Gag^M^ + gp120HA_2_, (**iii**) Gag^M^ + gp140HA_2_tr, (**iv**) Gag^M^. Scale bars represent 100nm. (**b**) VLPs were isolated from RK13 cells infected with MVA vaccines by gradient centrifugation and analyzed by Western blotting. (**c**) Relative ratios of Env:Gag in VLPs were determined by densitometry analysis of Western blots.

As shown previously, expression of Gag^M^ by cells transfected or infected with the DNA or MVA vaccines led to VLP formation (Figure 5). No differences were observed in the sizes of the VLPs (data not shown), suggesting that the Env chimeras had no effect on VLP size. Relative ratios of Env:Gag in VLPs isolated from the media using gradient centrifugation indicated that there were also no significant differences between VLPs containing gp150 or the chimeric Envs (Figure 5c). This was in line with the FACS data, where there was no indication that the spike density of the chimeric Envs was increased relative to that of gp150, as almost all the MAbs showed slightly higher levels of binding to gp150 than the chimeras (Figure 4). Only F105 showed increased binding to the chimeras as compared to gp150.

### 3.4. Immunogenicity Testing

**Figure 6:**
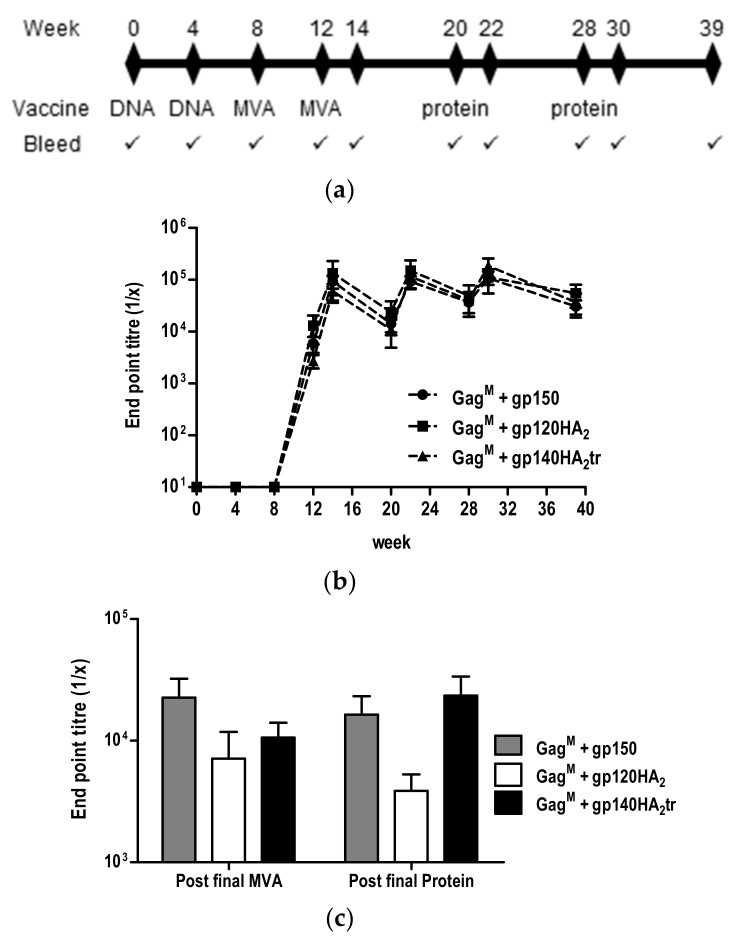
Vaccination schedule and characterization of binding antibodies induced in immunized rabbits. (**a**) Rabbit vaccination regimen. (**b**) Binding antibody titers in sera were quantified in an indirect ELISA using CAP256 SU gp140 trimers as the capture antigen. (**c**) Titers of binding antibodies to scaffolded CAP256 SU V1V2 loop at week 14 (post final MVA) and week 39 (post final protein).

To compare the ability of the different chimeric envelopes to elicit antibodies, rabbits were immunized with two doses of the DNA vaccines, followed by two doses of the matching MVA vaccines and, lastly, two boosts with soluble gp140 protein in AlhydroGel^®^ (InvivoGen, Toulouse, France)(Figure 6a).

Binding antibodies to the autologous gp140 antigen developed after the first MVA vaccination (week 8) and were maintained eleven weeks after the last protein boost for all three groups of rabbits (Figure 6b). MVA and protein vaccinations boosted the binding antibody titers, but no differences in binding antibody titers were observed between the vaccines expressing gp150 and the chimeric Envs. Similarly, no differences in the binding antibody titers to the scaffolded CAP256 SU V1V2 loop measured 2 weeks after the final protein boost (week 30) were detected (Figure 6c).

Serum samples collected at different time points were also analyzed for neutralizing antibodies in a pseudovirus assay using TZM-bl cells (Table 2). Neutralization of Tier 1A pseudovirus MW965.26 was observed after the first MVA inoculation in all three groups of rabbits with no clear differences in titers between the different groups. These neutralization titers significantly increased after the second MVA inoculation for all three groups of rabbits. However, differences were seen in the development of neutralizing antibodies to the Tier 1B 6644 pseudovirion between the group of rabbits that received vaccines expressing the gp120HA_2_ chimera and those receiving gp150 or the gp140HA_2_tr chimera. All five rabbits in the gp150 Env group (reciprocal serum dilution causing a 50% reduction of relative light units (ID_50_) 41 to 170), four out of five in the gp140HA_2_tr group (ID_50_ 89 to 158), and only one out of four in the gp120HA_2_ group (ID_50_ 83) developed neutralizing antibodies against 6644 after the final vaccination. Similarly, clear differences in the neutralization titers towards the autologous Tier 2 CAP256 SU pseudovirus were also seen between groups of rabbits that received vaccines expressing the gp120HA_2_ chimera (0/5 rabbits) and those receiving gp150 (4 / 5 rabbits) or the gp140HA_2_tr chimera (3 / 5 rabbits).

The bNAbs elicited to CAP256 SU Env during infection, like other V2bNAbs, recognized a lysine-rich region (residues 168 to 171) on the V2 apex of Env and also interacted with a glycan at N160 [15,21,28,29,30,31]. However, when rabbit sera were tested against CAP256 SU K169E pseudovirions, no differences in neutralization was observed compared to CAP256 SU (data not shown). Similarly, the sera did not neutralize two heterologous pseudoviruses where the VIV2 loops of BG505N332 and CAP84 had been replaced with that of CAP256 SU (data not shown). This indicated, as previously found for the gp150 Env [13], that the neutralizing antibodies in the sera of rabbits, vaccinated with the gp140HA_2_tr chimeric Env, did not target the V1/V2 region of the CAP256 SU pseudovirus.

The week 30 sera (Table 2, P2), from rabbits that showed Tier 2 neutralization against CAP256 SU, were tested against a global panel of ten HIV-1 Env pseudoviruses. Two of the four rabbits from the group that received DNA and MVA vaccines expressing gp150 Env, and all three of the rabbits from the group that received DNA and MVA vaccines expressing gp140HA_2_tr developed low titers of neutralizing antibodies against Clade A 398F1 pseudovirus (ranging between 1:20–29) (Table 3).

## 4. Discussion

In this study, we aimed to increase the expression of HIV Env on the surface of virus-like particles by exchanging either the entire gp41 region of the HIV envelope protein or just the TM + CT region with cognate sequences derived from influenza A H5 HA_2_ protein.

However, no differences in the ratios of Env:Gag were seen in VLPs isolated from cells transfected with DNA or infected with MVA vaccines expressing the chimeras compared to gp150, as determined by Western blotting. Furthermore, there was no indication from the Env MAb FACS data that the chimeric constructs increased Env spike density on the plasma membrane of HEK293T cells compared to gp150. This differed from studies carried out by other groups, where increased incorporation of HIV Env on the surface of Gag VLPs was seen for Env chimeras containing the TM + CT regions of Epstein–Barr virus, baculovirus, mouse mammary tumor virus, and influenza virus glycoproteins as compared to HIV gp160 [8,9,11,12]. It was possible in our case, however, that the modifications made to the HIV gp150 envelope protein already significantly increased the levels of Env in Gag VLPs to a level that could not be further increased by the replacement of the TM + CT regions with that of the influenza hemagglutinin protein. Various studies have shown that truncation of the HIV Env from gp160 to gp150 increases the levels of expression on the surface of virus particles [10,32]. In addition, the replacement of the native HIV Env leader sequence with other leader sequences has also been shown to increase expression [10,33,34]. The use of the flexible glycine linker sequence is also expected to prevent the shedding of the gp120 portion of the HIV Env trimer from the surface of the VLPs [23], resulting in higher levels of detection of gp120 on the surface of the VLPs.

Although there appeared to be no significant differences in the levels of expression of Env on the surface of cells between the gp150, gp140HA_2_tr chimera, and the gp120HA_2_ chimera, there did appear to be differences in the quaternary structure of the three proteins, as no binding of MAbs PG16, CAP256-VRC26.08, or PGT145 to the surface of cells expressing gp120HA_2_ was detected, and increased levels of binding of F105 was detected. MAbs PG16, CAP256-VRC26.08, and PGT145 bound a quaternary epitope on the V2 apex of the HIV Env trimer and could, therefore, be used to screen for antigens with a native, trimeric structure, whereas F105 bound to non-native trimers. These results indicated that the replacement of the HIV Env MPER with that of the HA_2_ negatively affected the quaternary structure of the envelope protein. While binding of the MAbs detecting natively folded Env was similar between gp150 and the gp140HA_2_tr chimera, gp150 showed a slightly superior presentation of bNAb epitopes for PGT128, PGT135, PG9, and 10E8 compared to the gp140HA_2_tr chimera. These data, combined with the increased affinity of F105 for the gp140HA_2_tr chimera, would suggest a slightly superior folding of gp150 compared to the gp140HA_2_tr chimera.

The altered quaternary structure of the gp120HA_2_ Env chimera resulted in the complete lack of autologous Tier 2 NAbs in the sera of rabbits vaccinated with DNA and MVA vaccines expressing this chimera. Live cell and FACS-based cell surface staining of cells expressing the gp140HA_2_tr chimera and gp150 showed that some of these envelope proteins were in a native, trimeric conformation. Most of the rabbits inoculated with DNA and MVA vaccines expressing the gp140HA_2_tr chimera or gp150 developed autologous Tier 2 NAbs, indicating that an HIV Env antigen with a native-like quaternary structure was required to elicit Tier 2 NAbs [35,36]. No Tier 2 NAbs were detected in the sera of rabbits vaccinated with VSV-GP expressing HIV gp140 fused to the VSV glycoprotein G [12]. This might also have been due to the poor quaternary structure of the VSV-GP-gp140:G chimera, as only very low levels of binding of the bNAb PG16 were seen to VSV-GP particles and to the surface of cells expressing the chimeras. MAbs 10E8 and 4E10, which bound to the MPER of HIV Env, also bound very poorly to these VSV-GP-gp140:G chimeras. Although other groups have constructed HIV-1 Env chimeras using the TM + CT regions of Epstein–Barr virus, baculovirus, mouse mammary tumor virus, and influenza virus glycoproteins that have shown increased expression on the surface of the cells and virus-like particles, they do not appear to have carried out assays to determine whether the HIV-1 Env chimeras were in a native, trimeric conformation. Our data and that of Bresk et al. (2019) indicated that chimeras, in which the TM + CT regions of HIV-1 Env were substituted with that of other viral glycoproteins, might not be presented in a native, trimeric conformation able to elicit neutralizing antibodies.

The DNA and MVA vaccines expressing the gp140HA_2_tr chimera or gp150 elicited high titers of binding antibodies to the V1V2 region of CAP256 SU and autologous Tier 2 NAbs. The binding antibodies elicited probably also bound to many other sites, but this was not confirmed as only the V1V2 region was tested in this study. However, there was no evidence that the fusion of gp140 to HA_2_ increased the density of Env or enhanced the immune response over truncated Gp150. These data confirmed the importance of characterizing the antigenicity of HIV-1 Env as a means of predicting Tier 2 autologous neutralization.

## Figures and Tables

**Table 1:** Live-cell staining of the envelope (Env) and Env chimeras, expressed on the surface of cells, with monoclonal antibodies (MAbs).

Antibody	Neutralization	Epitope	Native-Like Trimer	Live Cell Mapping *		
				gp150	gp120HA_2_	gp140HA_2_tr
PGT128	Broad	V3-glycan supersite	x	√	√	√
PGT135	Broad	V3-glycan supersite	x	√	√	√
447-52D	Narrow	V3-glycan	x	√	√	√
VRC01	Broad	CD4 binding-site	x	√	√	√
F105	Narrow	CD4 binding-site	x	√	√	√
PG9	Broad	V2 apex	x	√	√	√
PG16	Broad	V2 apex	Yes	√	X	√
PGT145	Broad	V2 apex	Yes	√	X	√
CAP256-VRC26.08	Broad	V2 apex	Yes	√	X	√
10E8	Broad	MPER	x	√	X	Weak

* Cells were infected with MVA vaccines expressing Gag^M^ + gp150, Gag^M^ + gp120HA_2_, or Gag^M^ + gp140HA_2_tr; or transfected with DNA vaccines expressing gp150, gp120HA_2_, or gp140HA_2_tr.

**Table 2:** Neutralizing antibody titers elicited in rabbits.

		Clade C Tier 1A					Clade C Tier 1B					Clade C Tier 1B					Clade C Tier 2					Control
		MW965.26					6644					1107356					CAP256 SU					MLV
		ID_50_ after:					ID_50_ after:					ID_50_ after:					ID_50_ after:					ID_50_ after:
Regimen	Rabbit	pre	M1	M2	P1	P2	pre	M1	M2	P1	P2	pre	M1	M2	P1	P2	pre	M1	M2	P1	P2	pre-wk30
DDMMPP gp150 Gag^M^	6826	<20	195	3426	5286	7840	<20	<20	85	45	170	<20	<20	32	<20	29	<20	<20	70	486	1294	<20
	6827	<20	119	3892	14601	2748	<20	<20	120	196	104	<20	<20	35	<20	<20	<20	<20	53	519	174	<20
	6828	<20	81	7239	664	4348	<20	<20	172	20	136	<20	<20	62	<20	21	<20	<20	<20	<20	<20	<20
	6830	<20	108	12641	20778	3920	<20	<20	299	172	143	<20	<20	40	<20	<20	<20	<20	60	332	296	<20
	6850	<20	<20	1999	1987	787	<20	<20	78	40	41	<20	<20	<20	<20	<20	<20	<20	<20	32	54	<20
	median	<20	108	3892	5286	3920	<20	<20	120	45	136	<20	<20	35	<20	<20	<20	<20	53	332	174	<20
DDMMPP gp120HA_2_ Gag^M^	6832	<20	<20	470	3594	1115	<20	<20	<20	30	<20	<20	<20	21	<20	<20	<20	<20	<20	<20	<20	<20
	6833	<20	166	82	96	211	<20	<20	<20	<20	<20	<20	<20	<20	<20	<20	<20	<20	<20	<20	<20	<20
	6834	<20	36	42	305	148	<20	<20	<20	21	<20	<20	<20	<20	<20	<20	<20	<20	<20	<20	<20	<20
	6835	<20	25	2410	1050	3680	<20	<20	130	74	83	<20	<20	<20	<20	23	<20	<20	<20	<20	<20	<20
	median	<20	31	276	678	663	<20	<20	<20	26	<20	<20	<20	<20	<20	<20	<20	<20	<20	<20	<20	<20
DDMMPP gp140HA_2_tr Gag^M^	6836	<20	35	8193	6134	1786	<20	<20	595	175	158	<20	<20	47	<20	<20	<20	<20	<20	<20	<20	<20
	6838	<20	97	1060	6524	3235	<20	<20	113	154	113	<20	<20	<20	<20	25	<20	<20	<20	<20	<20	<20
	6839	<20	53	578	8835	2578	<20	<20	32	262	89	<20	<20	<20	24	<20	<20	<20	<20	242	151	<20
	6840	<20	44	101	384	298	<20	<20	<20	<20	<20	<20	<20	<20	<20	<20	<20	<20	<20	103	44	<20
	6842	<20	87	NT	5783	2816	<20	<20	NT	112	128	<20	<20	NT	<20	<20	<20	<20	NT	171	220	<20
	median	<20	53	819	6134	2578	<20	<20	73	154	113	<20	<20	<20	<20	<20	<20	<20	<20	103	44	<20

The serum tested was taken two weeks post each MVA boost (M1 and M2) and two weeks post each protein inoculation (P1 and P2). The 50% neutralization titers were color-coded to reflect their potency range as follows: yellow = 20 – 100; orange = 100 – 1000; red = 1000 to 10 000; dark red = 10 000 – 100 000. Titers below 20 were considered to be negative for neutralizing activity and not color-coded. NT= not tested (in white). It should be noted that these data points M2, P1, and P2 for the vaccines expressing the CAP256 SU gp150 have been published previously and were included here for comparative purposes [13].

**Table 3:** Neutralizing antibody titers elicited in rabbits against a global panel of pseudovirions.

	Virion	X1632	398F1	25710	BJX2000	CE0217	CE1176	CH119	CNE8	CNE55	TRO.11	X2278	246F3
	Clade	G	A	C	CRF07	C	C	CRF07	CRF01	CRF01	B	B	AC
Group	Rabbit	ID_50_ at week 30											
DDMMPP Gag^M^ + gp150	6826	<20	<20	<20	<20	<20	<20	<20	<20	<20	<20	<20	<20
	6827	<20	21	<20	<20	<20	<20	<20	<20	<20	<20	<20	<20
	6830	<20	24	<20	<20	<20	<20	<20	<20	<20	<20	<20	<20
	6850	<20	<20	<20	<20	<20	<20	<20	<20	<20	<20	<20	<20
DDMMPP Gag^M^ + gp140HA_2_tr	6839	<20	22	<20	<20	<20	<20	<20	<20	<20	<20	<20	<20
	6840	<20	22	<20	<20	<20	<20	<20	<20	<20	<20	<20	<20
	6842	<20	24	<20	<20	<20	<20	<20	<20	<20	<20	<20	<20

The 50% neutralization titers were color-coded to reflect their potency range. Yellow = 20 – 100. Titers below 20 were considered to be negative for neutralizing activity and not color-coded.
